# PET-CT in the diagnosis of cardiac and pulmonary sarcoidosis

**DOI:** 10.1007/s00508-022-02129-5

**Published:** 2022-12-20

**Authors:** Marcus Brugger, Klemens Scheidhauer, Jochen Gaa

**Affiliations:** 1 Department of Internal Medicine I, University Hospital München rechts der Isar, Technical University of Munich, Munich, Germany; 2 Department of Nuclear Medicine, University Hospital München rechts der Isar, Technical University of Munich, Munich, Germany; 3 Department of Radiology, School of Medicine, University Hospital München rechts der Isar, Technical University of Munich, Munich, Germany

## Additional case report to the image

**Fig. 1:**
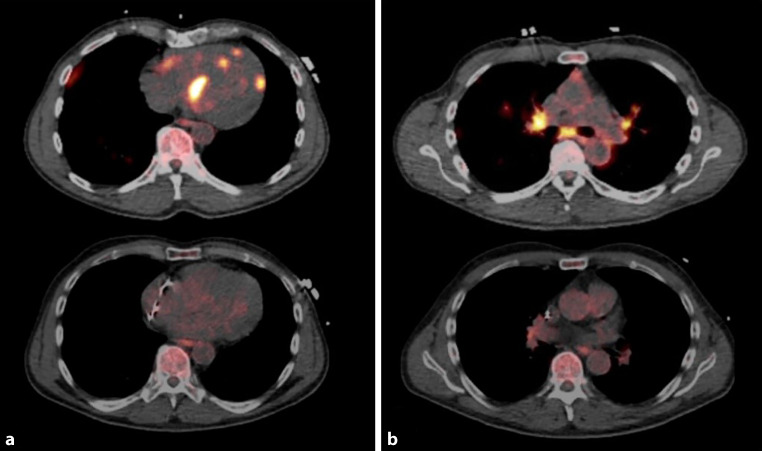
**a** Increased metabolic activity in PET/CT imaging with 332 MBq (F-18)-fluorodeoxyglucose in the left ventricular myocardium mainly basal anteroseptal, midventricular dorsal and the inferior papillary muscle. Right ventricular involvement is low (*top*). Remission after 5 weeks follow-up under immunosuppressive monotherapy with 50 mg prednisolone per day (*bottom*). **b** Bipulmonary increased metabolic activity mainly in the middle lobe and right upper lobe and additionally prominent mediastinal lymph nodes (*top*). Remission of metabolic activity due to immunosuppressive therapy (*bottom*)

A 65-year-old male was admitted to the hospital to further investigate broad complex tachycardia. Coronary heart disease was excluded previously. Subsequent cardiac magnetic resonance imaging revealed the typical picture of cardiac sarcoidosis and additional lung involvement in computed tomography (CT) scan. Left ventricular ejection fraction was preserved (LVEF 52%) and right ventricular ejection function was impaired (RVEF 32%). The transbronchial biopsy confirmed numerous granulomas containing giant cells without caseating necrosis in the lung parenchyma. In accordance, fluorodeoxyglucose positron emission tomography/computed tomography (FDG-PET/CT)-imaging (Fig. 1) showed bipulmonary granulomatous changes with increased metabolic activity, mainly in the middle lobe and right upper lobe and additionally prominent cervical and mediastinal lymph nodes. The cardiac involvement of the sarcoidosis was extensive with intensely increased metabolic activity in the left ventricle, most likely inducing broad complex tachycardia. An immunosuppressive monotherapy with 50 mg prednisolone per day was initiated [1, 2]. For secondary prophylaxis, an implantable cardioverter defibrillator (ICD) was implanted [3]. In the FDG-PET/CT follow-up after 5 weeks, the extensive involvement of the myocardium was completely regressive. Furthermore, there were minor fine nodular pulmonary changes on both sides without metabolic activity compared to the previous examination. Thus, multimodal noninvasive imaging can be helpful in detecting and determining the extent of cardiac and pulmonary sarcoidosis.
